# Applications of Highly Stretchable and Tough Hydrogels

**DOI:** 10.3390/polym11111773

**Published:** 2019-10-28

**Authors:** Zhen Qiao, Jesse Parks, Phillip Choi, Hai-Feng Ji

**Affiliations:** Department of Chemistry, Drexel University, Philadelphia, PA 19104, USA; zhen.qiao@drexel.edu (Z.Q.); jesse.james.parks@drexel.edu (J.P.); pjc329@drexel.edu (P.C.)

**Keywords:** highly stretchable, tough, hydrogel, polyacrylamide, chemical crosslinking, physical crosslinking, sensor, drug delivery, wound healing

## Abstract

Stretchable and tough hydrogels have drawn a lot of attention recently. Due to their unique properties, they have great potential in the application in areas such as mechanical sensing, wound healing, and drug delivery. In this review, we will summarize recent developments of stretchable and tough hydrogels in these areas.

## 1. Introduction

Due to the high water content and excellent biocompatibilities, hydrogels have been used for wound healing, tissue culture, tissue/cartilage replacement [1], scaffolds for cell growth [2], etc. When combined with electronics, hydrogels have been used to develop a variety of biomedical devices, including sensors [3,4,5,6,7,8,9,10,11,12,13], switchable micro patterns [14], self-oscillators [15], etc. Most hydrogels have relatively low tensile strength and low elasticity, which limits their application capacity in the biomedical field. In order to improve the mechanical properties of hydrogels, highly stretchable and tough hydrogels have been invented recently [16]. These highly stretchable and tough hydrogels generally consist of one or more gel networks of two or more polymer chains crosslinked both chemically (typically via covalent bond) and physically (typically via intermolecular interactions) [17,18]. Recently, we wrote a review on the synthesis and fundamental properties of highly stretchable and tough hydrogels [19]. In this review, we will summarize the applications of the most up-to-date highly stretchable and tough hydrogels for mechanical sensing, drug delivery, and wound dressing. This review is a part II, with a focus on the applications.

## 2. Highly Stretchable and Tough Hydrogels for Mechanical Sensing

Mechanical sensors are a class of sensors used to measure the mechanical properties of an object. One major approach of measurement is through piezoresistivity, which is the effect exhibited when there is a change in resistance due to applied pressure. Many of these piezoresistive sensors, also known as strain gauges, were made from a polyester base with wire or metallic foil. These sensors generally have a low stress limit and cannot be readily repaired when damaged. Highly stretchable, tough hydrogels have high stress limits and can self-heal over time. Mechanical hydrogel sensors are wearable, due to the high stress limit that they possess, i.e., their reasonable stretchability. When appraising the use of hydrogels in sensors, one must consider the gauge factor of the gel, the healing time and efficiency, and the mechanical property of hydrogels.

### 2.1. Gauge Factor

Gauge factor is the ratio of the change in resistance of the gel to the strain applied, which is defined as $=\frac{\Delta R/R}{\Delta L/L}=\frac{\Delta R/R}{\varepsilon }=1+2\nu +\frac{\Delta \rho /\rho }{\varepsilon }$, where *ε* is the strain, *L* is the change in length, *L* is the original length, *ν* is the Poisson’s ratio, *ρ* is the resistivity, *R* is the change in strain resistance, *L* is the unstrained resistance.

**Figure 1:**
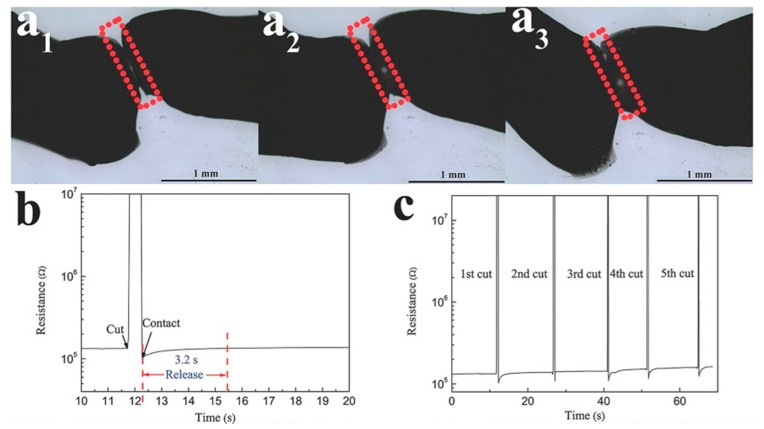
(**a1**–**a3**) Images of polyvinylalcohol (PVA)/single wall carbon nanotube (SWCNT) hydrogel healing at times 0, 30, 60 s, respectively, at room temperature. (**b**) Electrical healing process by measuring resistance with time under ambient conditions. (**c**) Cycles of cutting and healing of hydrogel. (Adapted from [21] Figure 2, with permissions from Wiley).

In 2015, Frutiger et al. [20] developed a soft strain sensor, by modifying a commercially available silicone, that exhibited a high stretchability (up to 700%), however, this elastomer tended to have low gauge factors (0.348 ± 0.11). In 2016, Cai et al. [21] developed a polyvinylalcohol (PVA)-based hydrogel with a single wall carbon nanotube (SWCNT) conductor in an attempt to create a highly stretchable gel with high gauge factor (Figure 1). The hydrogel exhibited a gauge factor of 0.24 at 100% strain and 1.51 at 1000% strain. The gauge factor was shown to have improved due to the addition of the SWCNT conductor, based on the gauge factors that were recorded of the gel without the SWCNT conductor (0.09 at 100% and 0.53 at 1000%).

**Figure 2:**
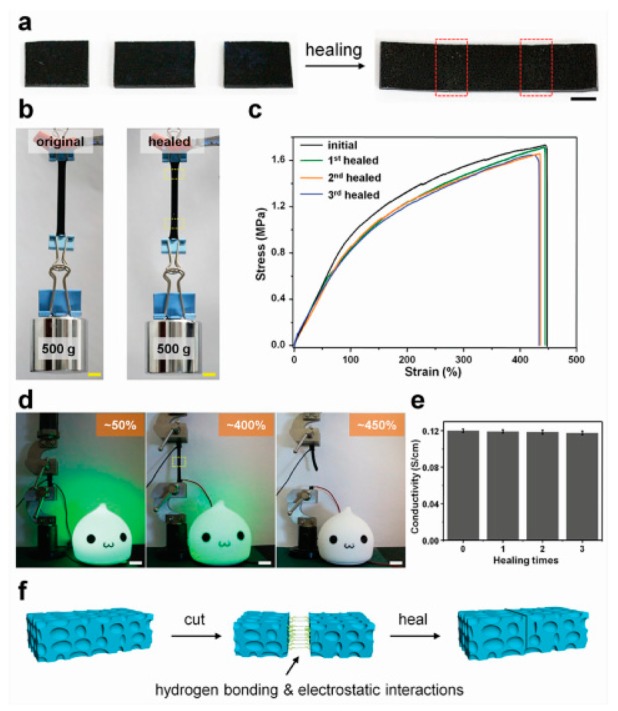
(**a**) Cutting and healing of polyacrylic acid (PAA)/polyaniline (PANI) hydrogel. (**b**) Hydrogel supporting ~500 g mass. (**c**) Strain percentage vs. stress over multiple healing cycles. (**d**) Electrical conductivity test of healed hydrogel with a green LED bulb. (**e**) Electrical conductivity graph over multiple healing cycles. (**f**) Illustration of hydrogel healing process. (Adapted from [22], Figure 3, with permissions from Wiley).

Wang et al. [22] determined that the gauge factor can be improved with a synthesized hydrogel with polyaniline (PANI) and polyacrylic acid (PAA) in 2018 (Figure 2). After cutting and healing (a), the hydrogel can hold a ~500 g mass (b). Strain percentage vs. stress over multiple healing cycles showed the gel had a gauge factor of 11.6 for up to 100% strain and 4.7 for strains above 100% (c). The gel showed the combination of PANI and PAA attributes to the high stretchability and conductivity of the gel. Electrical conductivity test showed that the hydrogel healed with a green LED bulb could stretched more than 400% before breaking (d), and the gel can be healed multiple times without losing the conductivity (e). The hydrogel healing process was also discussed (f).

**Figure 3:**
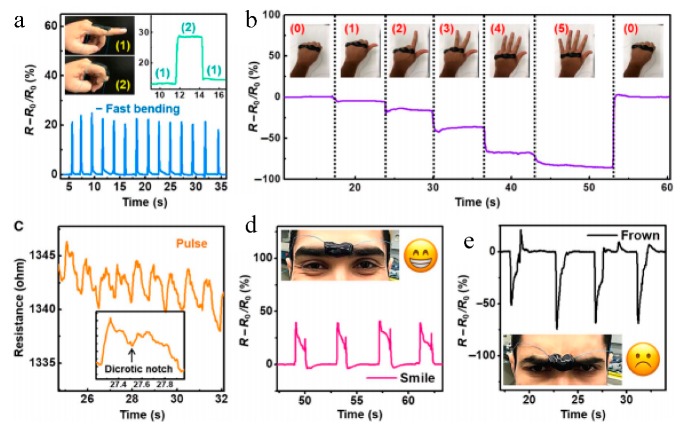
(**a**–**e**) Change in resistance of PVA/MXene hydrogel due to various gestures and facial expressions. (Adapted from [23], Figure 4, with permissions from AAAS).

At a similar time in 2018, Zhang et al. [23] proposed the use of MXenes in hydrogels to improve the sensor performances (Figure 3). The PVA/MXene hydrogel demonstrated high stretchability and a high gauge factor. The recorded gauge factor was 25 at 40% strain. The conductive MXene filler increased strain sensitivity and mechanical properties of the PVA gel, due to MXene nanosheets having an abundance of surface functional groups. These functional groups can alter the surfaces to become negatively charged and hydrophilic.

### 2.2. Healing Time and Efficiency

Hydrogel sensors are able to heal from any deformations that occur. This includes the self-healing of the gel’s appearance, electrical recovery, and mechanical recovery. The inability to heal in any of those ways would negatively impact the use of the hydrogel as a sensor.

The PVA/SWCNT hydrogel was able to heal its appearance partially in 30 s and completely within 60 s at room temperature, with no scarring left on the gel. While the gel may have only partially healed, the PVA/SWCNT gel manages an electrical healing time of 3.2 s [21].

Liu et al. [24,25] prepared a PVA-based hydrogel that is self-healing and self-adhesive. They used polydopamine (PDA) to assist in self-healing and adhesiveness, and achieved complete self-healing in 250 milliseconds under ambient temperature. Similarly, Zhang et al. [26] utilized MXenes to increase the sensitivity of PVA-based hydrogel. The PVA/MXene hydrogel showed an ‘instantaneous’ healing time. MXenes, having numerous surface functional groups, contributes to the hydrogen bonding found in PVA, which explains the incredible healing time.

### 2.3. Stretchability

If a sensor is to be wearable, it should generally be highly stretchable to ensure comfort for the wearer. Shao et al. [25] formulated an ionic hydrogel based on polyacrylic acid (PAA) and aluminum ions. The gel achieved a fracture strain of 2952%, showing that the gel is ultra-stretchable. The above-mentioned PVA/MXenes hydrogels was able to achieve a stretchability of 3400%. Most recently, in 2019, Yang et al. developed a PVA/Borax gel. The biocompatible hydrogel has the ability to stretch up to a strain of 5000%.

Table 1 summarizes other hydrogel-based mechanical sensors that have been developed from a variety of groups.

## 3. Highly Stretchable and Tough Hydrogels for Wound Healing

Although hydrogels are already being utilized for wound healing, many of these commercial hydrogels have flaws. For instance, commonly used in surgery to create a fibrin clot, fibrin glue has issues with weak adhesion strength and the risk of transfer of blood diseases [52,53]. Fibrin glue TISSEEL [54] is vulnerable to debonding. Polyethylene glycol–based adhesives like COSEAL, although it has good adhesion properties and effectively prevents leaking of blood from vessels, carries poor mechanical properties and induces swelling [55]. Similarly, cyanoacrylate-based adhesives, super glues, are hampered by their poor biomechanical compatibility [56]. Despite being the strongest class of tissue adhesives, these adhesives are cytotoxic and poor with wet surfaces, making it difficult to be applicable to wound healing [57]. In terms of self-healing, a commercial carboxymethyl hydrogel only healed 27% in a week and 70% in 10 days [58]. With these flaws, researchers have looked for alternative solutions and methods to create hydrogels better suited for wound healing with efficient healing time and effective adhesion strength.

### 3.1. Healing Properties

When hydrogels are examined for their uses in wound healing, the healing time is a critical factor. Healing time is the necessary time needed for a wound to heal. In 2013, Sakai et al. developed a simple yet effective method that allows a highly stretchable gel to in situ gelate when a solution is poured onto a wound [58]. This solution, containing a PVA derivative with phenolic hydroxyl properties (PVA-Ph), glucose oxidase (GOx), and horseradish peroxidase (HRP), allows the hydrogelation to occur as quickly as five seconds. This PVA-Ph hydrogel was able to heal 77% of the initial wounds in seven days and 96% within 10 days, which is more effective than the previously mentioned commercial carboxymethyl hydrogel. Le et al. combined dopamine-modified four-armed poly (ethylene glycol) (PEG) and poly (sulfamethazine ester urethane) (PSMEU) to better control physical and mechanical properties [59]. The PEG-PSMEU hydrogel was tested on longitudinal cuts to measure healing time. These wounds completely closed after a week. These developed hydrogels also had other healing characteristics. Consequently, Qu et al. reported that loading the quaternized chitosan/Pluronic^®^ F127 (QCS/PF) gel with curcumin results a tunable antioxidant ability [60]. Ballance et al. reported that the addition of cyclodextrin in a polyacrylamide gel increases mechanical strength [61]. More importantly, this hydrogel demonstrated antibacterial properties when treated with quinine. The gel’s improved stretchability resulted in a greater release of quinine, leading to successfully inhibit the growth of *E. Coli*. Liu et al. reported the combination of PEG-D4 with Laponite forms an injectable gel that degrades nontoxically [53]. In fact, many of the developed hydrogels are applicable on tissue skin unlike the previously mentioned commercial adhesives.

### 3.2. Adhesion Strength

To be applicable to wound healing, gels must have great adhesion strength. Adhesion strength is how a polymer sticks and bonds to surfaces. When adhesion strength is measured, multiple tests are usually done on a variety of surfaces. To compare with human skin, porcine skin was commonly used. Qu et al. increased the adhesion strength of 4.4 kPa to 6.1 kPa on porcine skin with the increase of Pluronic^®^ F127 (PF127-CHO) [60]. This strength was comparable to that of a fibrin glue adhesive. Li et al. developed tough adhesives, the toughest of which had an adhesion strength of 83 kPa on porcine skin [62]. The adhesion occurred in a few minutes, lending itself to more uses, unlike cyanoacrylate, which hardens upon contact with tissue skin. Han et al. added polydopamine (PDA)-intercalated clay nanosheets to a PAM hydrogel to make it more adhesive [63]. While the hydrogel only has an adhesion strength of 28.5 kPa on porcine skin, it showed 120 kPa when measured on glass. When adhered to the glass slides, this hydrogel could support a load of 500 g. Most recently, Wang et al. made a tyrosine hydrochloride gel with the adhesion strength of 453 kPa on pigskin [64]. As time progressed, the adhesion strength has rapidly improved in hydrogels.

### 3.3. Additional Mechanical Properties

He et al. reported that the introduction of a microgel to enhance adhesiveness and improve mechanical strength in its poly (*N*-isopropylacrylamide) microgel/polyacrylic acid-polyacrylamide-polydopamine (MR/PAAc-PAM-PDA) hydrogel [65]. Fukao et al. combined bioceramic hydroxyapatite (HAp) with double network hydrogels to increase mechanical strength and create a structure similar to bone tissue [66]. Guvendiren et al. reported the integration of 3,4-dihydroxy-l-phenylalanine (DOPA) to the gel increases cohesive and adhesive properties [67]. Unlike healing time, self-healing time refers to the time needed for the hydrogel to recover. In 2018, Chen et al. crosslinked oxidized sodium alginate–dopamine (OSA-DA) and polyacrylamide (PAM) to withstand large deformations and efficiently self-heal [68]. OSA-DA-PAM hydrogel was able to recover 80% within only six hours. Using the dynamic coupling reaction of tyrosine hydrochloride catalyzed by enzymes, Wang et al. developed a gel that completely self-healed within a day in 2019 [64]. The hydrogel was 25% healed in four hours and 68% in 12 h, showing much progress.

Table 2 summarizes other hydrogel-based wound dressings that have been developed from a variety of groups.

## 4. Highly Stretchable and Tough Hydrogels for Drug Delivery

Highly stretchable, wearable hydrogels have a high potential in use for delivering drugs. In 2013, Zhang et al. formulated an enzyme-incorporated hydrogel made from PAAm and alginate [69]. This gel maintained homogenous throughout, even after being stretched 6.67 times its original size. The gel was put through a 48-h intensive straining and washing process that concluded with no leakage of proteins in the gel. Over a week of time, enzymes in the hydrogel maintained most of their initial activity when stored at room temperature. Any loose, free counterparts in solution, however, lost activity significantly. Park et al. created a hydrogel made from poly (methacrylic acid/ethylene glycol dimethacrylate) and Fe_3_O_4_ in 2015 [70]. The composite microcapsules in the gel are biocompatible, responsive to pH, and magnetic, which makes it appropriate for drug delivery. The amount of drug, in this case doxorubicin chloride (DOX), released was 43.8% at pH 2 compared to 9.5% at pH 7. In the same year, Di et al. [71] worked on a tensile strain-triggered drug delivery device. The gel was made of poly (lactic-*co*-glycolic acid) nanoparticles and Dragon Skin 30, which is a soft, strong, stretchy silicone rubber. The nanoparticles serve as drug delivery depots, while the Dragon Skin works for loading tensile strain. The amount of insulin release saturated after 10 testing cycles at 50% strain with two second intervals. With an interval of four hours, the same amount of insulin was released across several stretching events. Lin [35] demonstrated that when stretched, a polyacrylamide-based gel with alginate and titanium wire accommodates drug diffusion without any breakages or leaking, with a drug diffusion coefficient of 3 × 10^−10^ m^2^ s^−1^. A biocompatible, stretchable, and robust material was fabricated by Liu et al. [48]. The hydrogel is a polyacrylamide-based gel with alginate and polydimethylsiloxane. The design that Liu put forward could be programmed with desirable functionalities by designing the circuits in the cells and structures and patterns of the hydrogel.

## 5. Conclusions

Highly stretchable and tough hydrogels can be implemented in multiple different fields, such as mechanical sensors. Properties important to making a good mechanical sensor, such as gauge factor, self-healing, and having strong mechanical properties, can be seen within these hydrogels. Highly stretchable and tough hydrogels, with great adhesion strength and healing times, can be utilized for wound healing. The mechanical properties of tough, highly stretchable hydrogels can be used for drug delivery.

## Figures and Tables

**Table 1:** The properties of highly stretchable and tough hydrogels.

Gel	Problems of Traditional Gels that the New Gel Tried to Fix	Design Strategy of the New Gel in the Paper	Gauge Factor (Strain %)	Healing Time and Efficiency	Mechanical Properties	Year	Ref.
PVA/SWCNT	No sensing properties over 100% strain, Low Gauge Factor	Introduce SWCNT to increase stretchability, gauge factor, and recovery	0.24 (100%) 1.51 (1000%)	Electrical Healing: 3.2 s Appearance: 30–60 s Self-Healing Efficiency: ~98%	No change in sensor properties after 1000 cycles at 700% strain Excellent Sensing Performance	2016	[21]
PVA/Graphene	No sensing properties over 100% strain, Low Gauge Factor	Introduce Graphene to increase stretchability, gauge factor, and recovery	0.92 (1000%)	-	Excellent Sensing Performance	2016	[21]
PVA/Silver Nanowire	No sensing properties over 100% strain, Low Gauge Factor	Introduce Silver Nanowire to increase stretchability, gauge factor, and recovery	2.25 (1000%)	Silver nanowire is easily oxidized by air and water	Excellent Sensing Performance	2016	[21]
Aromatic Polyamic Acid Salt (PAAS) Hydrogel	Poor Mechanical Properties, Preparation is toxic	Prepare in an environmentally friendly way, Adding p-PDA/s-BPDA enhance mechanical properties	-	Self-healed within 1 min at room temperature	Mechanical stress of 500 kPa at 1350% strain, Storage Modulus of 5 × 10^5^ Pa	2019	[27]
DCh/PPy/PAA	Low Conductivity, Sensitivity, Mechanical Recovery	Create a mechanically/electrically self-healing hydrogel with pressure/extension sensitivity	-	Mechanical Recovery: 2 min 90% Electrical Recovery: 30 s	Conductivity increases with strength of compression on Hydrogel	2017	[28]
PVA/PVP/Fe^3+^	Low Mechanical Properties, Self-healability, sensitivity	Fabricate a conductive, elastic, self-healing, and strain-sensitive hydrogel	0.478 (200%)	Self-healing within 5 min, and self-recovery within 30 min	Mechanical Strength of 2.1 MPa of tensile stress	2017	[29]
PVA/PDA	Low Detection Ranges and sensitivity	A low-modulus PVA hydrogel that is self-healing, PDA makes the hydrogel self-adhesive	-	Self-Healing in 250 ms at ambient temperature	High Sensing Performance in the ranges of Ultralow (0.1%) to High (500%) Strain	2018	[24]
PEDOT:SL/PAA	Not wearable, Insensitive to pressure/strain Can freeze at subzero temperatures	PEDOT:SL improves softness and elasticity-promotes strain sensitivity	7 (100%)	-	Stretched to 7 times original length, recovers with negligible residual strain	2019	[30]
PAAm/Graphene	Poor mechanical consistence and electrical conductivity	Hydrogel acts as potential scaffold for neuronal growth	9 (30%)	-	Conductivity:5.4 × 10^−5^ S/cm	2018	[31]
PAA/PANI	Self-healing electronics have low durability and stretchability	PANI-based self-healing electronic composite with high stretchability and electrical conductivity	11.6 (Within 100%) 4.7 (Over 100%)	Electrical Conductivity Healing Efficiency: 88.2% in 5 min Mechanical Healing Efficiency: 24.3% in 5 min	Stretchability up to 400% Electrical Conductivity of 0.12 S/cm	2018	[22]
PAAm/LiCl	Ionogels have lower conductivity than hydrogels	Soft, stretchable electrical devices integrating a conductive hydrogel	0.84 (40%)	-	Conductivity: 10.39 ± 0.31 S/m	2017	[32]
PAA/Graphene/Fe^3+^	Low stretchability, self-healing, mechanical properties	Covalent bonds -strong, stable network for the hydrogel, Reduced graphene oxide and ions are highly sensitive	0.31 (100%) 1.32 (500%)	Recovered nearly 100% initial conductivity	Resistance: 5.8 kΩ Strength: ~300 kPa at 45% strain Tensile Strength: ~400 kPa at 300% strain	2018	[33,34]
PAA/Al^3+^	Poor mechanical properties, Require adhesives	Ions allow high sensitivity to large and subtle motions	5.5 (100%) 7.8 (2000%)	Healing efficiency of 88% at 20 min and 92% at 30 min	Ultra-stretchability with a 2952% fracture strain, Compression Performance: 95% strain without fracture Toughness: 5.60 MJ/m^3^	2018	[25]
Dopamine/Talc/PAAm (DTPAM)	Low stretchability and recoverability	Polydopamine-modified talc particles uniformly disperse in PAAm—Enhance mechanical properties/adhesiveness	0.125 (100%) 0.693 (1000%)	Appearance healed after 30 min at room temperature	After healing, can still be stretched over 800% Strongly adhesive	2018	[34]
PAAm/Alginate	Low mechanical robustness and stretchability	PAAm and alginate form a ‘tough’ hydrogel that has a high stretchability and fracture toughness	-	-	Fracture Toughness of ~9000 J/m^2^ Fatigue Fracture of 53 J/m^2^ Cycle 1000: Constant resistance to high stretching	2016 2017	[35,36]
PAAm/Alginate/CaCl_2_	Desired properties lost below freezing point of water	Gel soaked in 30 wt % CaCl_2_ retains stretchability/toughness/conductivity at below 0 °C	-	-	Fracture Toughness of ~5000 J/m^2^	2018	[37]
PAAm/Alginate Optical Fibers	Fragile against external strain, Low mechanical strength	Make a tough hydrogel, which has high stretchability and mechanical strength	-	-	Fracture Energy of ~9000 J/m^2^ Can be elongated to 700% strain	2016	[38]
PAMPS/PAAm Double Network Gel	Single network hydrogels showed poor mechanical properties, Fatigue Damage under low cyclic load	Double Network hydrogels have outstanding mechanical properties	-	-	Average Toughness ~3358 J/m^2^, Fracture Energy 3779 J/m^2^, Fatigue Threshold 418 J/m^2^	2018	[39]
PVA/PAAm	Low stretchability and sensitivity	Adhesive Wrinkled microarchitectures and interconnected ridges increase contact area	-	-	Stretchability up to 500%, Response time of 150 ms, Sensitivity of 0.05 kPa^−1^ at 0 to 3.27 kPa	2018	[40]
AAm/2-hydroxyethylacrylate/Liquid Gallium	Low sensitivity, limited stretchability, and poor stability	Use liquid metals as soft fillers in hydrophilic polymer networks to make highly stretchable, force-sensitive hydrogels	-	-	Tensile Strain ~1500%, Compressive Sensitivity of 0.25 kPa	2019	[41]
PAA/PANI	Limited by fragile and weak properties, like low flexibility	Highly Stretchable PAA/PANI hydrogel	0.60 (0–800%) 1.05 (800–1130%)	-	Tensile Deformation: 1160% strain Sensing Range: 0 to 1130%	2018	[42]
PVA/MXene	Low sensitivity	MXenes have high conductivity and strain sensitivity. MXenes improve mechanical properties	2, 0 wt % MXene (40%) 25, 4.1 wt % MXene (40%)	Instantaneous Self-Healing	Stretchability of 3400% Conformability and adhesive to various surfaces, including human skin	2018	[23]
PAAm/Alginate/Eutectic Gallium	Low Conductivity, Stretchability, High Power Consumption	Eutectic Gallium is highly conductive and used in a known tough hydrogel	-	-	Sensitivity of 100 Pa, can be rehydrated to most of its initial weight (>85%) after 30 drying/soaking cycles	2018	[43]
PAAm/Agar/LiCl	Low stretchability, Opaque, Poor Mechanical Strength	Conductive, Excellent mechanical properties, stretchability, and sensitivity, Transparent	1.8 (1100%)	-	Stretchability over 1600%, Tension Strength: 0.22 MPa, Compression Strength: 3.5 MPa	2019	[44]
PDMS/AAm/NaCl	Capacitance and resistance are affected by stretch, bend, and pressure	Low Cost Materials and methods	-	-	Ionic Resistivity of 0.06 Ω	2017	[45]
PAAm/LiCl	Low Sheet Resistances and transparency, Brittle	Used as an ionic conductor	-	-	Can operate with over 1000% areal strain Elastic Modulus of 12 kPa	2016	[46]
PAAm/LiCl/Silicone	LED-based systems are limited by low ultimate strain	Fabricate a hyperelastic light-emitting capacitor (HLEC), using a hydrogel	-	-	Stretches to >480% strain	2016	[47]
PAAm/Alginate/PDMS	Low mechanical robustness and compatibility	Hydrogel–Elastomer Hybrid that is stretchable, robust, and biocompatible	-	-	-	2017	[48]
PNAGA-PAMPS/PEDOT-PSSa	Conductive Hydrogels (CHs) are mechanically weak and brittle	PNAGA hydrogels demonstrate high strength, thermoplasticity, and self-healability	-	Self-healed after 3 h in a plastic syringe immersed in a 90 °C water bath	0.22–0.58 MPa tensile strength, 1.02–7.62 MPa compressive strength, 817–1709% breaking strain	2017	[49]
PVA/CNF	Low sensitivity, stretchability, self-healability, and transparency	Highly sensitive, stretchable, and autonomously self-healing ionic skin—biocompatible	-	Spontaneously Self-Healed in 15 s	Highly Transparent—Transmittance as high as 90%, Modulus of 11.2 kPa, Elongation Rate of 1900%	2019	[50]
PVA/Borax	Low stretchability, self-healing, water retention, biocompatibility	PVA and Borax: biocompatible/highly stretchable/easily dissolvable in aqueous solution/have good mechanical performance	-	Self-healed 10 times without affecting electrical conduction of gel	Can be stretched to strains over 5000%	2019	[51]

PVA—Polyvinyl Alcohol; SWCNT—Single-Wall Carbon Nanotube; p-PDA—p-Phenylenediamine; s-BPDA—Biphenyltetracarboxylic dianhydride; DCh—Double-bond Decorated Chitosan; PPy—Polypyrrole; PAA—Polyacrylic Acid; PVP—Polyvinylpyrrolidone; PDA—Polydopamine; PEDOT:SL—Poly (3,4-ethylenedioxythiophene): Sulfonated Lignin; PAAm—Polyacrylamide; PANI—Polyaniline; PAMPS—Poly (1-acrylanmido-2-methylpropanesulfonic acid); AAm—Acrylamide; PDMS—Polydimethylsiloxane; PNAGA-PAMPS—Poly (N-acryloyl glycinamide-co-2-acrylamide-2-methylpropanesulfonic); PEDOT-PSS—Poly (3,4-ethylenedioxythiophene)-poly (styrenesulfonate); CNF—Cellulose Nanofibril.

**Table 2:** The properties of highly stretchable and tough hydrogels for wound healing.

Gel	Problems the Gel Tried to Fix	Design Strategy	Healing Time	Adhesion Strength (kPa)	Conclusion	Year	Ref.
Tyrosine Hydrochloride Gel	Poor mechanical properties and self-healing properties	Use dynamic coupling reaction to improve adhesion and self-healing properties	Self 4 h: 25% 12 h: 68% 24 h: 100%	Pigskin: 453 Glass: 265 Stainless Steel: 265 PTFE:329	This gel exhibited great self-healing abilities.	2019	[64]
MR/PAAc-PAM-PDA Hydrogel	Limited adhesion strength	Introduce MR to enhance adhesion and improve mechanical strength	-	40 after 60 s	Stretch up to 660% at a tensile strength of 110 kPa. Good self-healing properties	2019	[65]
PEG-D4 Laponite Gel	Weak adhesion, poor biomechanical compatibility.	Add Laponite to PEG-D4 to promote bioactivity increase adhesion and mechanical properties	-	Wt % Laponite 0%: 3.5 1%: 7 2%: 8	Injectable gel degrades nontoxically. Laponite increases adhesion strength.	2014	[53]
DOPA gel	Unknown effects of DOPA on cohesion and adhesion	Integrate DOPA to the gel to test cohesive and adhesive properties	-	-	Become worse in adhesion at higher pH levels.	2008	[67]
OSDA-DA-PAM Hydrogel	Poor mechanical property, lack of tissue adhesiveness	Crosslink OSA-DA and PAM chains to withstand large deformations	Self 6 h: 80%	-	Improved mechanical properties, Useful self-healing ability.	2018	[68]
PEG-PSMEU Hydrogel	Nuclease degradation, lack of membrane permeability	Combine PEG and PSMEU to better control mechanical properties	longitudinal cutaneous wounds: healed in 7 days	90	Higher copolymer concentration leads to higher adhesion strength.	2018	[59]
QCS/PF Gel	Questionable reliability of dressing materials on wound	Increasing content of PF127-CHO increases adhesive strength	-	6.1	Good blood-clotting ability	2018	[60]
Cur-QCS/PF Gel	Questionable reliability of dressing materials on wound	Loading the gel with Cur will result tunable antioxidant ability. Greater release rate	-	-	Better healing from greater release rate, Better Collagen levels after 15 days	2018	[60]
PAMPS/PAM DN Gel	Unable to be firmly fixed onto bones by glues.	Inducing bioceramic HAp on the gel surface for robust bonding to bone tissues.	-	-	Bonelike structure by controlling HAp crystal orientation. Bonded to bone tissue.	2017	[66]
PAM-cyclodextrin Gel	Nonstretchable PAM gel has intrinsic brittleness	Combine cyclodextrin acrylate to increase strain	-	-	Quinine inhibited growth of E. Coli. Stretched 16 times original	2018	[61]
PVA-Ph Hydrogel	Challenge in situ formation of hydrogel wound dressings	HRP-catalyzed reaction so the gel can form in situ on wound	7 days: 77% 10 days: 96%	-	Gelated as quickly as 5 s. Easily pour onto wound. Retained mechanical properties.	2013	[58]
PDA-clay-PAM Hydrogel	Weak adhesive materials and poor mechanical properties	Adding PDA-intercalated clay nanosheets will make it more adhesive	-	Glass: 120 Ti:80.8 PE:80.7 Porcine Skin:28.5	Strong adhesiveness, High stretchability, Good candidate for delicate surgical adhesive.	2017	[63]
Tough adhesives	Commercial adhesives have weak adhesion vulnerable to debonding	Fabricate family of tough adhesives that can adhere to wet surfaces.	-	On beating porcine heart: 83	Hemostatic dressing possible, High adhesion energy High matrix toughness	2018	[62]

PTFE: Polytetrafluoroethylene; PNIPAM: Poly (*N*-isopropylacrylamide); MR: PNIPAM microgel; PAAc-PAM-PDA: Poly (acrylic acid)-poly (acrylamide)-poly (dopamine); PEG-D4: Dopamine-modified four-armed poly (ethylene glycol); DOPA: 3,4-Dihydroxy-l-phenylalanine; OSA-DA: Dopamine-grafted oxidized sodium alginate; PEG: Poly (ethylene glycol); PSMEU: Poly (sulfamethazine ester urethane); QCS: Quaternized chitosan; PF: Pluronic^®^ F127; Cur: Curcumin; PAMPS: Poly (2-acrylamido-2-methylpropanesulfonic acid); DN: Double Network; HAp: Hydroxyapatite; SN: Single Network; PVA: Polyvinyl alcohol; Ph: Phenolic Hydroxyl; HRP: Horseradish peroxidase; GOx: Glucose oxidase; PDA: Polydopamine; PAM: Polyacrylamide; PAA: Polyallylamine.
